# POCUS Evaluation in Acute Kidney Injury

**DOI:** 10.24908/pocus.v6i2.14775

**Published:** 2021-11-23

**Authors:** Vanessa A Hoytfox, Brittney C Ward, Emily J Cox, Kang X Zhang

**Affiliations:** 1 Providence Internal Medicine Residency Spokane, Providence Health Care Spokane, WA United States of America; 2 Providence Medical Research Center, Providence Health Care Spokane, WA United States of America

**Keywords:** hydronephrosis, ultrasound, POCUS, renal colic

## Abstract

Acute kidney injury is a common clinical problem encountered in general internal medicine. The evaluation of acute kidney injury is mainly driven by the patient’s clinical history, physical exam, and laboratory investigation including urinalysis and urine sediment examination. Point of care ultrasound (POCUS) may be a useful tool to help clinicians to narrow and/or prioritize differential diagnosis in patients presenting with acute kidney injury. Here we present a case of a 67-year-old male presenting with dysuria, fevers, and flank pain along with elevation in serum creatinine who was admitted with concern for acute kidney injury secondary to complicated urinary tract infection. Subsequent kidney POCUS of the kidneys and bladder showed bilateral anechoic fluid collection within the kidney sinus with dilated calyces suggestive of bilateral hydronephrosis, most likely due to a new diagnosis of benign prostatic hyperplasia. This case demonstrates the use of POCUS-obtained valuable diagnostic information and subsequent therapeutic management for this patient presenting with suspected acute kidney injury.

## Description of the case

**Figure 1:**
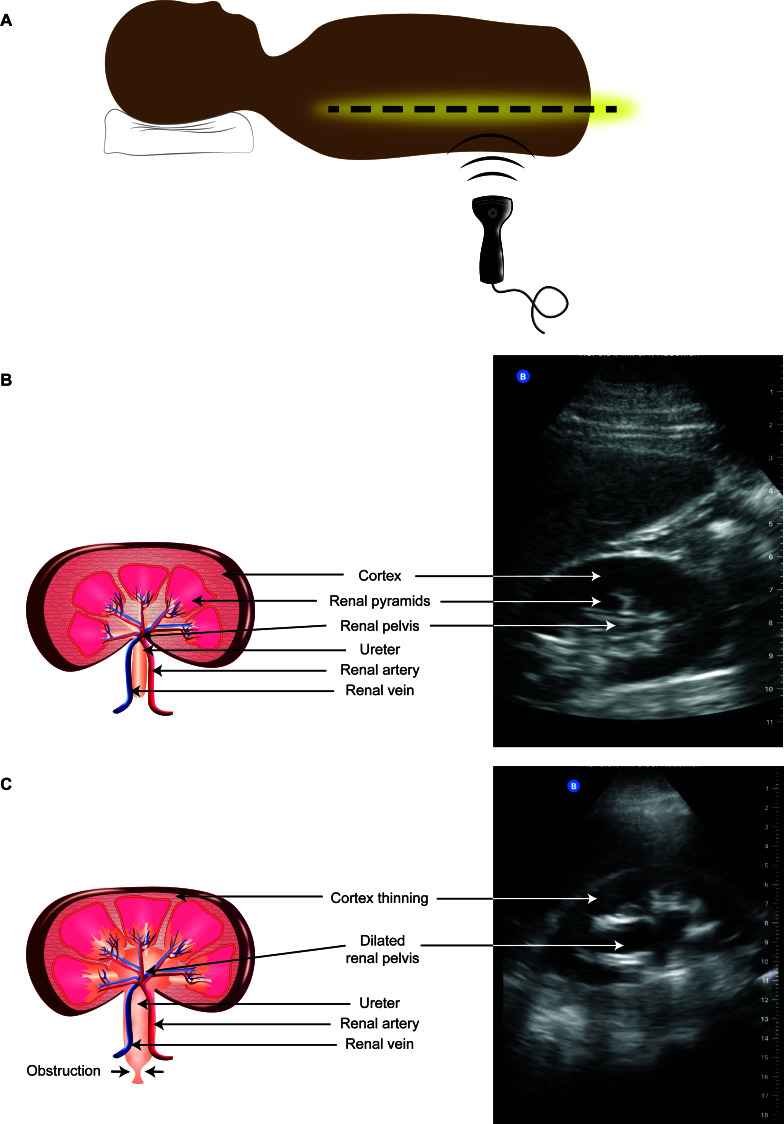
POCUS evaluation in acute kidney injury. Image A. With the patient supine, place a low-frequency probe (probe marker pointing cephalad) at the mid-axillary line (dotted line) just above the costal margin, with the ultrasound beam directed into retroperitoneal space. The longitudinal l view of the kidney should be in view. The probe should then be rotated clockwise 90 degrees to obtain the transverse view (image not shown). Image B. Representative image of normal right kidney in the longitudinal view with structures labeled. Note the normal kidney architecture with the non-dilated kidney pelvis and preserved kidney parenchyma (cortex, pyramids). Image C. Representative image of hydronephrosis of the right kidney in the longitudinal view with structures labeled. Note the abnormality within the urinary collecting system including the central dilation of the kidney pelvis (anechoic) and calices which appear anechoic within the hypoechoic narrowed kidney cortex.

A previously healthy 67-year-old male presented to the emergency department after 4 days of bilateral flank pain consistent with kidney colic. The pain was associated with dysuria, fevers, urinary hesitancy, and frequency. He was afebrile and hemodynamically stable on initial presentation. Physical exam was significant for lower abdominal and left costovertebral angle tenderness. Relevant labs showed a leukocytosis of 22,000 cell/µL with 13% bands and creatinine of 2.61 mg/dL without a known prior baseline. Urinalysis was positive for pyuria, blood, and nitrite. The patient received broad-spectrum antibiotics and was admitted for presumed acute kidney injury secondary to pyelonephritis. The admitting resident team performed POCUS evaluation of the kidneys and found an anechoic collection bilaterally within the kidney sinus along with dilated calyces (Figure 1). Additional color flow Doppler was applied to the anechoic space and confirmed the absence of a vascular component. These findings supported the determination that kidney injury was likely multifactorial due to obstructive nephropathy in addition to pyelonephritis. A Foley catheter was placed immediately for bladder decompression. Comprehensive kidney and bladder ultrasonography was performed by radiology within 2 hours of POCUS exam. This confirmed severe bilateral hydronephrosis as well as mobile echogenic debris and thickened bladder wall suggestive of cystitis. Left kidney size was 12.8 cm and right kidney size was 14.3 cm. Post-void residual bladder volume was ~400 ml and enlarged prostate was visualized. Urology was consulted and the patient required bilateral ureteral stent placement. The most likely diagnosis was pyelonephritis complicated by hydronephrosis secondary to enlarged benign prostatic hyperplasia. The patient’s clinical symptoms markedly improved and creatinine improved to 2.46 mg/dL. He was discharged with oral antibiotics and outpatient urology follow-up.

This case demonstrates that POCUS of the kidneys and bladder can assist providers in visualizing complications of urinary obstruction and thus guide further diagnostic imaging and decision-making. Evaluation of acute kidney injury is a common clinical problem encountered in general internal medicine, and post-obstructive acute kidney injury is often considered as part of differential diagnosis. In this case, the detection of anechoic fluid collection in the kidney sinus by POCUS shifted the diagnostic momentum as it prompted timely management and evaluation of obstructive uropathy. 

The American College of Radiology (ACR) Appropriateness Criteria for acute pyelonephritis does not recommend imaging in uncomplicated cases of pyelonephritis 1. However, in complicated cases, ultrasonography offers a low-risk and rapid imaging acquisition modality 1. In a multicenter, randomized trial comparing initial imaging methods (POCUS vs radiology ultrasound vs abdominal CT) in patients with suspected nephrolithiasis, initial ultrasonography led to significantly lower 6-month cumulative radiation exposure in both ultrasonography groups compared with the CT group, without significant differences in diagnostic accuracy, treatment outcomes, or re-admissions 2. 

While it does not replace the need for comprehensive imaging, the use of POCUS in the evaluation of acute kidney injury allows quick and accurate ascertainment of the need for further diagnostic/therapeutic interventions. Previous studies have shown that POCUS can quickly detect hydronephrosis with a sensitivity of 77-90% and specificity of 71-96% 3, 4, 5, 6. Emergency physicians can correctly identify hydronephrosis via POCUS with an accuracy of 81% and a positive predictive value (PPV) of 91% compared to CT scans 5. Ultrasound performed by emergency physicians has shown comparable results to CT in detection of severity of hydronephrosis, in which hydronephrosis detected by emergency physicians using POCUS had a PPV of 88% and likelihood ratio of +2.91 7. Skill acquisition is a minimal barrier: indeed, accuracy of POCUS is not significantly limited by training level or scanning experience^3^, ^8^ and even untrained emergency physicians can learn to accurately detect or rule out hydronephrosis after a 2 day, 16-hour training course 8, 9. POCUS is commonly performed by rural physicians with various levels of POCUS training in New Zealand in the evaluation of urinary retention to identify hydronephrosis with a sensitivity of 90% and specificity of 96% 6. In a study evaluating the comparative diagnostic accuracy of hydronephrosis with POCUS versus CT scan, research showed that physicians with as little as 2 weeks of POCUS experience were able to detect hydronephrosis with 70% sensitivity and 73% specificity 10, 11.

This case illustrates that POCUS can guide focused advanced imaging such as comprehensive ultrasound of kidneys and bladder performed by radiology or computed tomography of the abdomen and pelvis. Data to date indicate that POCUS is fast, accurate, and is an easy skill to acquire and train. Thus, integrating POCUS in the evaluation of acute kidney injury may decrease time to intervention, avoid needless radiation exposure, control associated costs, and perhaps reduce length of stay without variation in quality of diagnostic accuracy. 

## Statement of ethics/consent approval

HIPAA authorization was obtained from the patient to permit publication of this case report. The Providence St. Joseph Health Institutional Review Board determined that this case report did not constitute research or require Institutional Review Board review.

## Disclosures

The authors have no disclosures to report.
